# Spatial and Functional Organization of Pig Trade in Different European Production Systems: Implications for Disease Prevention and Control

**DOI:** 10.3389/fvets.2016.00004

**Published:** 2016-02-04

**Authors:** Anne Relun, Vladimir Grosbois, José Manuel Sánchez-Vizcaíno, Tsviatko Alexandrov, Francesco Feliziani, Agnès Waret-Szkuta, Sophie Molia, Eric Marcel Charles Etter, Beatriz Martínez-López

**Affiliations:** ^1^Le Centre de coopération internationale en recherche agronomique pour le développement (CIRAD), UPR Animal and Integrated Risk Management (AGIRs), Montpellier, France; ^2^Center for Animal Disease Modeling and Surveillance (CADMS), Department of Medicine & Epidemiology, School of Veterinary Medicine, University of California Davis, Davis, CA, USA; ^3^Animal Health Department, Complutense University of Madrid, Madrid, Spain; ^4^Bulgarian Food Safety Agency, Sofia, Bulgaria; ^5^Istituto Zooprofilattico Sperimentale dell’Umbria e delle Marche, Perugia, Italy; ^6^Institut National Polytechnique-Ecole Nationale Vétérinaire de Toulouse (INP-ENVT), Toulouse, France; ^7^Epidemiology Section, Department of Production Animal Studies, Faculty of Veterinary Science, University of Pretoria, Onderstepoort, South Africa

**Keywords:** network analysis, community, movements, risk-based surveillance, swine, infectious diseases

## Abstract

Understanding the complexity of live pig trade organization is a key factor to predict and control major infectious diseases, such as classical swine fever (CSF) or African swine fever (ASF). Whereas the organization of pig trade has been described in several European countries with indoor commercial production systems, little information is available on this organization in other systems, such as outdoor or small-scale systems. The objective of this study was to describe and compare the spatial and functional organization of live pig trade in different European countries and different production systems. Data on premise characteristics and pig movements between premises were collected during 2011 from Bulgaria, France, Italy, and Spain, which swine industry is representative of most of the production systems in Europe (i.e., commercial vs. small-scale and outdoor vs. indoor). Trade communities were identified in each country using the Walktrap algorithm. Several descriptive and network metrics were generated at country and community levels. Pig trade organization showed heterogeneous spatial and functional organization. Trade communities mostly composed of indoor commercial premises were identified in western France, northern Italy, northern Spain, and north-western Bulgaria. They covered large distances, overlapped in space, demonstrated both scale-free and small-world properties, with a role of trade operators and multipliers as key premises. Trade communities involving outdoor commercial premises were identified in western Spain, south-western and central France. They were more spatially clustered, demonstrated scale-free properties, with multipliers as key premises. Small-scale communities involved the majority of premises in Bulgaria and in central and Southern Italy. They were spatially clustered and had scale-free properties, with key premises usually being commercial production premises. These results indicate that a disease might spread very differently according to the production system and that key premises could be targeted to more cost-effectively control diseases. This study provides useful epidemiological information and parameters that could be used to design risk-based surveillance strategies or to more accurately model the risk of introduction or spread of devastating swine diseases, such as ASF, CSF, or foot-and-mouth disease.

## Introduction

With 146 million pigs and a yearly production of about 22 million tons of carcass weight, the European Union (EU) is the world’s top exporter and the second biggest producer of pig meat after China (1). However, several transboundary animal diseases (TADs), such as African swine fever (ASF), classical swine fever (CSF), or foot-and-mouth disease (FMD), are of permanent risk of introduction or reintroduction in the EU swine industry (2, 3). Given the devastating impact outbreaks of such diseases can have on farmers, society, and EU countries economy, the European Commission strengthened the need of preparedness at both national and international levels to mitigate diseases risks and impacts (4).

Epidemic models are increasingly used to evaluate and inform disease surveillance and control policies (5, 6). As animal trade play a key role in the spread and control of most of TADs (7, 8), it is essential to include trade movement patterns to more realistically and accurately simulate the spatiotemporal spread of diseases and the effectiveness of control measures (9, 10). Since Regulation (EC) no. 1760/2000 of the European parliament, data on pig trade movements are registered at a farm level and daily scale in EU member countries. The full trade networks can be integrated in epidemic models to produce more realistic disease spread simulations [e.g., Ref. (11–13)]. However, considering the amount of data available, modeling transmission through full networks is computationally challenging and time-consuming, which would limit the usefulness of such models in a crisis period.

Different methodologies can be used to simplify and incorporate the major properties of pig trade patterns in epidemic models. Previous studies mostly used statistics on shipments rates, shipment distances, and mixing patterns between production types (14–17). Others included statistics on network topology (18, 19), as it has been shown that disease spread is sensitive to the topological structure of the contact network (20, 21). These statistics come from country specific data, expert opinions, or from countries with similar production systems (14, 16, 17). However, it is not clear how the parameters from one country can be translated to other areas (22), and few data are available for some specific production systems, such as outdoor or small-scale production systems (17, 23). Moreover, different production systems might coexist within a country, but their specific trade patterns might be hidden when computing statistics at country level. Community detection algorithms have been used to detect groups of premises that tend to trade together (24–26). They could be useful to identify different production systems within a country and better characterize their specific trade organization.

The objective of this paper was to fill part of those knowledge gaps by unraveling the functional and spatial organization of pig trade in the EU. Our aim was particularly to characterize and compare the trade structure and patterns in different pig production systems, including small-scale and extensive systems, for which scarce information is available so far. Results would be useful to better inform surveillance and control strategies as well as to more realistically parameterize disease spread models, particularly for TADs and other swine diseases with high economic impact, such as porcine respiratory and reproductive syndrome (PRRS).

## Materials and Methods

### Study Area

Four countries were selected to represent the diversity of European pig production systems: Bulgaria, France, Italy, and Spain. Spain and France are the second and third producers of pig meat in the EU, with intensive production systems, i.e., large-scale high-density indoor herds, concentrated mostly in Cataluña, Murcia, and Bretagne (1, 27). Italy is the seventh producer in the EU with intensive farming concentrated in the northern regions but also with high number of semi-intensive, medium, and small farms (1). In Bulgaria, such as other Eastern European countries, pigs are mostly reared by small producers (SP), mostly, for self-consumption (28, 29). Beside these systems, several regions have preserved traditional extensive production systems involving local breeds that are reared outdoor for the production of high-quality cured meat. Such systems are observed in south-central Spain, in south-west and central France, in south-central Italy, in the French and Italian Mediterranean islands of Corsica and Sardinia, and in the Eastern mountains in Bulgaria (30–33).

### Data Collection, Selection, and Preparation

Data on pig movements and premises characteristics were obtained from national databases, through Bulgarian Food Safety Agency (BFSA) in Bulgaria, the professional database of swine (La Base de Données Professionnelle Porcine – BDPORC) in France, the Istituto Zooprofilattico Sperimentale dell’Umbria e Marche (IZS-UM) in Italy, and the Ministry of Agriculture, Food and Environment (MAGRAMA) in Spain, under the appropriate confidential data transfer agreements. Registration of pig movements is mandatory in these countries since, at least, 2009. The year 2011, which was common in all databases, was retained for the analysis. Because of the dead-end characteristics of slaughterhouses, these premises were excluded from the analysis.

The premises characteristics available were the type of production, the premise size, the type of housing system (except for Bulgaria), the geographical coordinates, as well as the pig company number (only for France). In Bulgaria, pig farms were classified as East Balkan pigs (EBPs), SP (pigs kept for own consumption), Type B farms (TB = medium-size, low biosecurity level), Type A farms (TA = medium-size, high biosecurity level), or industrial farms (IND = large size, high biosecurity level) (28, 29). For France, Italy, and Spain, pig premises were categorized into seven distinct types: multipliers (MU: premises that produce breeding stocks and semen), farrowing farms (FA), farrow-to-finishing farms (FF), finishing farms (FI), SP, trade operators (TR), and unknown premise type (UP). FA included farms which produce piglets until 3 or 25 kg. FI included farms which buy piglets (at 3 or 25 kg) and produce either 25 kg piglets or fattening pigs. For Italy, farrowers and farrow-to-finishers could not be distinguished in the database and were thus both typed as FA. SP were defined as those who produce pigs for self-consumption in Spain, those who have no more than four fattening pigs and produce pigs for self-consumption in Italy (34), and farms with no more than four pigs in France. All farms that were not SP were considered as commercial farms. Trade operators included traders, collection centers, markets, fairs, and stop points. For those farms with no available coordinates, the centroid location (i.e., latitude and longitude) of the smallest geographical administrative unit available (village or municipality) was used. The main characteristics of the study area and study pig industries are presented in Table 1.

**Table 1:** **Description of pig industry in Bulgaria, France, Italy, and Spain in 2011**.

Country	Area (km^2^)	Road density (km/km^2^)	No. of premises	Premise type^a^ (%)							% outdoor
				MU	FA (IND)	FF (TA)	FI (TB)	SP	UP (EBP)	TR	
Bulgaria	110,944	0.36	28,729	NA	0.21	0.48	6.44	92.54	0.33	NA	NA
France	551,000	1.77	22,014	2.63	6.53	28.04	42.58	7.88	12.12	0.22	15.1
Italy	301,302	0.32	138,645	0.02	15.36	NA	9.85	71.12	3.48	0.17	26.4
Spain	505,954	1.50	92,389	0.95	5.09	31.53	20.03	40.77	1.21	0.42	19.8

*^a^For all countries: SP, small producer; for Bulgaria only: IND, industrial (large size, high biosecurity level farm); TA, type A farm (medium-size, high biosecurity level); TB, type B farm (medium-size, low biosecurity level); EBP, East Balkan pigs; for other countries: MU, multiplier; FA, farrowing farm; FF, farrow-to-finish farm; FI, finishing farm; UP, unknown type of premise; TR, trade operator; NA, not applicable/not available*.

Information on trade movements for all countries included the date of the movement, the unique identifier (ID) of the source and destination premises, and the number of pigs moved.

For each country, directed and weighted yearly networks were built, the nodes being all pig premises of the study areas, even those that were not trading pigs during the study period. Movement data were aggregated over the study period and a direct link was drawn whenever a shipment of pigs occurred between the corresponding premises. Two weights $W_{ij}^{\text{A}}$ and $W_{ij}^{\text{B}}$ were attributed to the link according to the number of pig batches and the number of pigs moved from premise *i* to premise *j* during the study period, respectively. The premises were considered as “active” if they moved pigs during the study period.

### Data Analysis

#### Descriptive Statistics

For each country, descriptive statistics were first generated including the number of active premises (i.e., premises that sent or received pigs in 2011), yearly shipment rate, shipment distance (i.e., Euclidean distance – in kilometers – covered for the shipment), and shipment size. The influence of premise type on these parameters was investigated by plotting their distribution per premise type pair.

The contact patterns between premises of different types were characterized by computing the normalized proportion of shipments per premise type pair. For each premise type pair (A,B), a relative shipment rate was calculated first $R_{\text{AB}}=S_{\text{AB}}/N_{\text{B}}$, where *S*_AB_ denotes the number of shipments from premise type A to B and *N*_B_ the number of premises of type B in the dataset. The relative shipment rates were then normalized to obtain a proportion of shipments received by premises of type B among all shipments sent by premises of type A, i.e., $\prod_{s}(\text{A,B})=R_{\text{AB}}/\left(\sum_{i}R_{\text{A}i}\right)$ (14).

#### Network Topology

For each country, pig trade networks were characterized in terms of (i) *network size*: number of nodes and number of links; (2) *network strengths*: weights of the links; and (3) *network cohesiveness*: percentage of isolates, density, local clustering coefficient, average path length, and sizes of the giant (i.e., the largest) strongly (GSC) and weakly (GWC) connected components. Descriptions of the network terminology used in this paper are outlined in Table 2 and are based on the definitions provided by Wasserman and Faust (35) and Robinson and Christley (36).

**Table 2:** **Network analysis glossary of terms interpreted in the context of pig movements**.

Parameter	Definition	Reference
Average path length	Average number of steps along the *shortest paths* for all possible pairs of nodes, i.e., the number of premises a premise has to trade through, on average, to reach any other premise. It measures the efficiency of infection flow on the network	(37)
Degree (*k*)	Number of contacts from and to a specific premise. When direction is taken into account, the ingoing and outgoing contacts are separated: the *out-degree* is the number of contacts originated from a specific premise, i.e., the number of premises receiving pigs from this premise; the *in-degree* is the number of contacts with direction to a specific premise, i.e., the number of premises that sent pigs to this premise. Nodes with high in-degree are more likely to acquire infection, whereas nodes with high out-degree are more likely to pass infection	(35)
Degree distribution *P*(*k*)	Probability distribution of the *degrees* over the whole network. In several networks, the degree distribution displays a power-law tail *P*(*k*) ~ *k*^−γ^, where the exponent γ is a constant. The tail of this distribution reflects the presence of *hubs*, which are nodes that have much higher contacts than the majority of the other nodes	(38)
Density (*D*)	Proportion of links that are present in the network compared to all possible links, calculated by the equation: *D* = 2*L*/*N*(*N* − 1). A value of 0 means that there are no links and 1 that all theoretically possible links are present. It informs about the speed at which infection may diffuse among nodes	(35)
Diameter	The largest *geodesic distance* in the network, i.e., the greatest number of links in the *shortest path* between two nodes	(35)
Components	Regions of the network where every node can be reached from every other node, either via directed paths (strong components) or ignoring the direction of the links (weak components)	(39)
Isolate	A node that did not send or receive pigs during the study period	(35)
Links (*L*)	A directed connection between two nodes representing pigs moved between two pig holdings	(35)
Local average clustering coefficient of the network	Average of local *clustering coefficient* over all nodes. The *clustering coefficient* of a node is the number of triangles (3-loops) that pass through this node, relative to the maximum number of 3-loops that could pass through the node. It indicates the likelihood that any two nodes with a common neighbor are themselves connected. Direction of the links is ignored and isolated nodes are not included in the averaging	(37)
Nodes (*N*)	Pig premises (farms, traders, etc.)	(35)
Shortest path	Number of links in the shortest possible walk from a node to another. It is also called *geodesic distance*	(35)
Weight (${\text{W}}_{\text{ij}}^{\text{A}}$ and ${\text{W}}_{\text{ij}}^{\text{B}}$)	The strength of a link. Two weights were considered in the present study to represent the amount of pig batches ${\text{W}}_{\text{ij}}^{\text{A}}$ and of pigs ${\text{W}}_{\text{ij}}^{\text{B}}$ moved from premise *i* to premise *j* during the study period. This parameter is used to better detect community structures	(35)

Each network was also assessed for scale-free and small world properties. To determine whether the networks were scale-free, a power-law distribution [*P*(*k*) ~ *k*^−γ^ with *k* being the degree and γ the power law scaling parameter] was fitted to the in- and out-degree distributions of each network, using statistical approaches described by Clauset et al. (40). The networks were considered to fit a power-law distribution if the *p*-value of the Kolmogorov–Smirnov test was higher than 0.05 (40). The degree distributions were visualized on log–log plots, with a straight line on such plots being suggestive of a power-law distribution (38). To assess if the networks exhibit small-world topology, their clustering coefficient and average path length were compared to those of 100 Erdös–Renyi random networks of equivalent size (same number of premises and of links). Observed networks were classified as small-world if they demonstrate at least sixfold increase in the clustering coefficient and decrease in average path length, in comparison to the analogous random network (41).

#### Trade Communities

Trade communities were identified using the “Walktrap” algorithm, a flow-based approach, with links weighted on the number of pig batches moved. The general idea of this approach is that random walkers following the links on the network tend to get “trapped” into densely connected parts corresponding to communities (42). The algorithm was applied on the whole networks. To check if the communities correspond to groups of premises with a shared common activity pattern, the ratio between the average weight of the links inside communities *w*_c_ and the average weight of the intercommunity links, *w*_ic_ were computed and compared between networks (43).

The largest communities (from a minimum of five to a maximum of 15) were selected based on the distribution of community sizes. These largest communities were mapped and their spatial extent was estimated. They were typed according to the proportion of premises by production type (commercial/small-scale farms) and housing system (indoor/outdoor). Their topology and functional organization were finally investigated by computing several descriptive and global network statistics.

All analyses were conducted in R (44) using the “igraph” package for network analysis (45).

## Results

### Descriptive Statistics

Descriptive statistics of the pig shipments are presented in Table 3. Shipment rates were generally quite low with a median <1–6 ingoing and 3–8 outgoing shipments per active premise per year (Table 3). Heterogeneity was observed between premises and between types of premises, with particularly high rates of incoming shipments for trade operators in France (Figure S1 in Supplementary Material). Median shipment distances varied from 3 km (Bulgaria) to 44 km (France) (Table 3). The premise type mostly sending pigs over long-range distances (>200 km) were industrial and type A farms in Bulgaria, multipliers in France and Spain, and trade operators in Italy (Figure S2 in Supplementary Material). Median shipment sizes varied from 4 (Bulgaria) to 220 pigs (Spain, Table 3). Shipment sizes tended to be higher when the pigs were sent to industrial farms in Bulgaria and to finishing farms in France, Italy, and Spain (Figure S3 in Supplementary Material). Pig batches sent to SP tended to be of small size and to come from local source (median number of pigs moved: 3, 3, 2, and 3 pigs; median shipment distance: 1, 21, 15, and 22 km in Bulgaria, France, Italy, and Spain, respectively).

**Table 3:** **Descriptive statistics of pig shipments in four European countries in 2011 (IQR, interquartile range)**.

Country	No. of active premises^a^ (%)	No. of ingoing shipments per active premise		No. of outgoing shipments per active premise		Euclidean shipment distance (km)		Shipment size (No. of pigs)	
		Median (IQR)	Max	Median (IQR)	Max	Median (IQR)	Max	Median (IQR)	Max
Bulgaria	1,349 (4.5)	1 (1–1)	121	3 (1–7)	107	3 (1–32)	433	4 (2–21)	1,750
France	12,454 (56.6)	6 (3–9)	2,838	8 (3–18)	324	44 (18–88)	811	21 (6–135)	9,286
Italy	50,553 (36.5)	1 (1–1)	1,106	4 (1–17)	1,174	17 (7–41)	1,033	5 (2–152)	2,804
Spain	27,339 (29.6)	2 (1–5)	1,375	3 (1–11)	310	37 (13–81)	988	220 (30–500)	13,950

*^a^Premises that sent or received pigs in 2011*.

**Figure 1:**
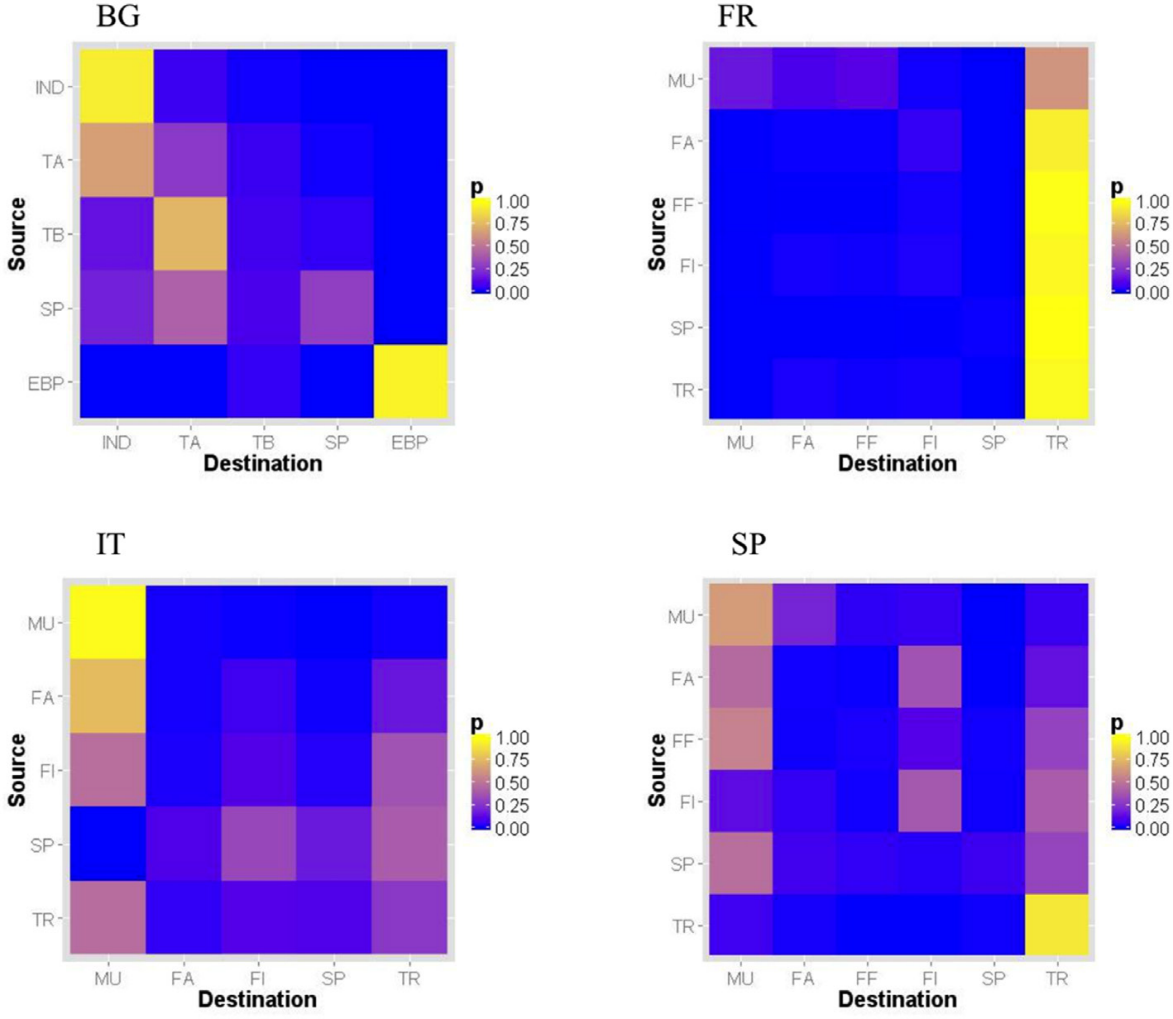
**Normalized relative proportion of pig shipments per premise type pair in Bulgaria, France, Italy, and Spain in 2011 [premise types: for all countries: SP = small producer; for Bulgaria only: IND = industrial (large size, high biosecurity level farm); TA = type A farm (medium-size, high biosecurity level); TB = type B farm (medium-size, low biosecurity level); EBP = East Balkan pigs; for other countries: MU = multiplier; FA = farrowing farm; FF = farrow-to-finish farm; FI = finishing farm; UP = unknown type of premise; TR = trade operator]. Unknown premise type not shown**.

Different mixing patterns by premise types were observed according to the country (Figure 1). In Bulgaria, industrial and EPB farms tended to trade with premises of the same type, whereas Type A farms tended to be intermediate between Type B and industrial farms. Multipliers tended to send pigs to multipliers, farrowing and farrow-to-finishing farms in France and Spain, whereas they were more likely to send pigs to multipliers only in Italy. Trade operators tended to be intermediate between farms and other trade operators in France and Spain, whereas they also tended to send pigs to multipliers and producers in Italy.

### Network Topology

Descriptive statistics of the pig trade networks are presented in Table 4. Presence of a high proportion of SP tended to increase the percentage of isolated premises. Indeed, most of SP did not report any pig movement in 2011 (95.7, 96.7, 63.6, and 91.3% of SP in Bulgaria, France, Italy, and Spain, respectively). Networks with a lot of commercial farms (France and Spain) tended to be denser and to have higher average degree (Tables 4 and 5). Italy presented the highest path length, clustering coefficient, and the largest GWC, and Bulgaria the smallest for all these statistics (Tables 4 and 5).

**Table 4:** **Descriptive statistics of pig trade networks in four European countries in 2011**.

Country	No. of nodes	No. of links	Median ${\text{W}}_{\text{ij}}^{\text{A}}$ (IQR)	Max ${\text{W}}_{\text{ij}}^{\text{A}}$	Median ${\text{W}}_{\text{ij}}^{\text{B}}$ (IQR)	Max ${\text{W}}_{\text{ij}}^{\text{B}}$	% isolates	Density (×10^−5^)	GSC size	GWC size
Bulgaria	28,729	1,127	1 (1–1)	107	3 (2–6)	42,970	95.3	0.1	2	172
France	22,014	29,487	1 (1–4)	88	59 (19–213)	32,820	43.4	6.1	74	12,083
Italy	138,645	58,193	1 (1–1)	77	3 (2–9)	64,570	63.5	0.3	69	46,403
Spain	92,389	42,362	1 (1–2)	111	200 (25–714)	105,300	70.4	0.5	49	21,723

*$W_{ij}^{\text{A}}$ and $W_{ij}^{\text{B}}$ are the links weights, i.e., the number of pig batches and pigs moved from premise *i* to premise *j* during the study period, respectively. GSC and GWC are the giant strongly and weakly connected components*.

**Table 5:** **Topological statistics of four European pig trade networks in 2011**.

Country	No. of nodes	No. of links	CC	AvPL	(*k*)	γ_o_	γ_i_	Max CC^Sim^	Min AvPL^Sim^
Bulgaria	28,729	1,127	0.051	1.3	0.08	NS	5.0	0.0000	1.03
France	22,014	29,487	0.096	4.5	2.68	2.9	2.8	0.0004	24.36
Italy	138,645	58,193	0.108	11.2	0.84	2.1	3.9	0.0000	1.69
Spain	92,389	42,362	0.052	4.2	0.92	4.1	3.5	0.0002	1.78

*CC is the clustering coefficient, AvPL is the average path length, (*k*) is the average degree, and γ_o_ and γ_i_ are the power-law scaling parameters for out- and in-degree distributions, respectively, NS means that the Kolmogorov–Smirnov test rejected the power law model as a plausible model for the degree distribution (*p* < 0.05), CC^Sim^ and AvPL^Sim^ are the clustering coefficients and average path length of 100 simulated Erdös–Renyi random networks of equivalent size*.

**Figure 2:**
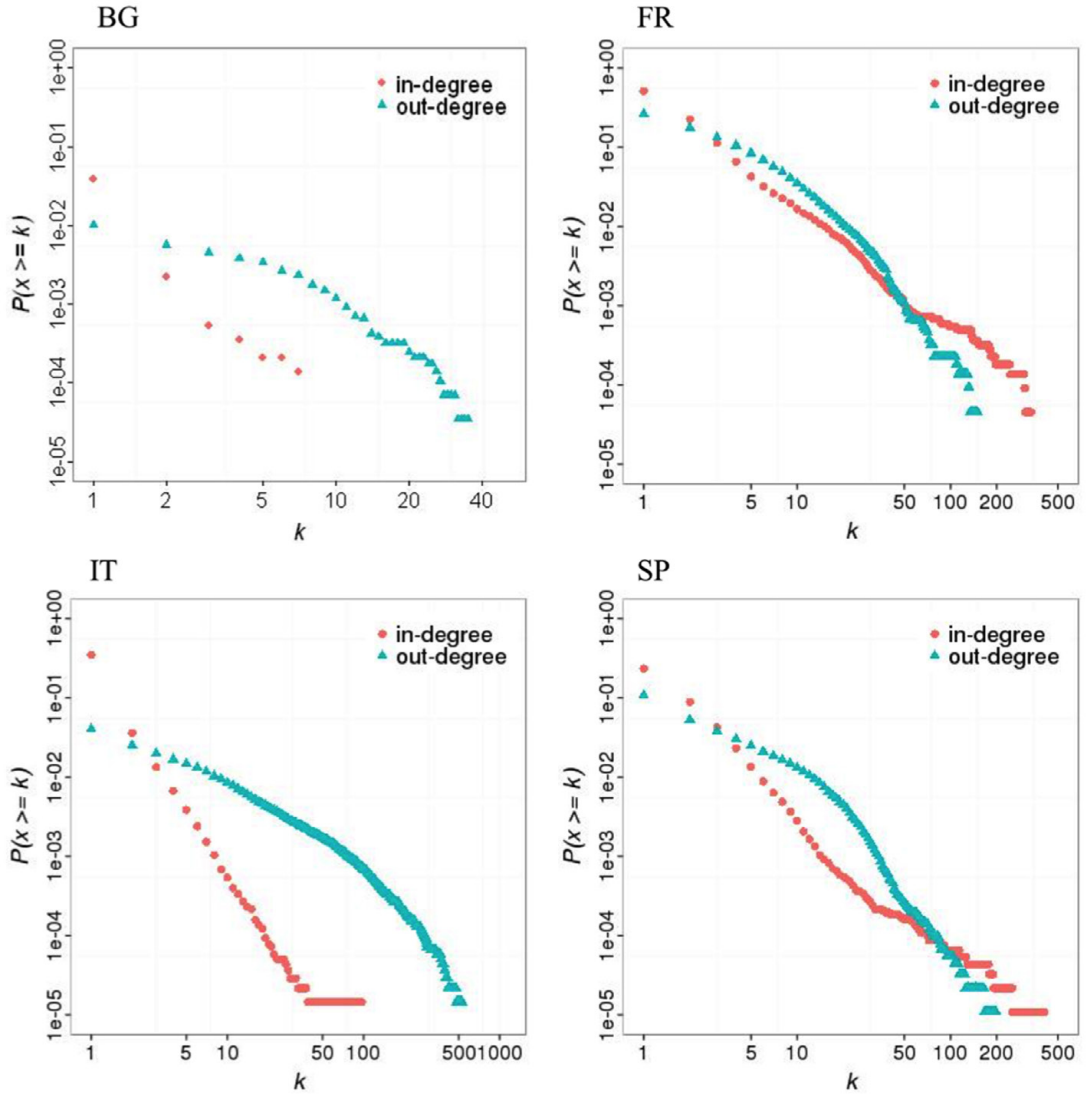
**Cumulative distribution of in- and out-degrees in four European pig trade networks in 2011 (BG: Bulgaria; FR: France; IT: Italy; SP = Spain)**.

All networks exhibited scale-free topologies (except the Bulgarian network for the out-degree distribution), with power law scaling parameters comprised between 2.1 and 5 (Table 5). These features can be observed on the in- and out-degree distributions which are broad (Figure 2). Results also show a clear asymmetry in receiving and sending activities (Figure 2; Figure S4 in Supplementary Material). In countries with a lot of SP (Bulgaria and Italy), premises tended to receive batches from a small number of premises but send them to a large number of premises. Conversely, in countries where commercial pig farms dominate (France and Spain), trade operators tended to receive batches from a large number of premises, assemble them, and moved then pigs to fewer premises.

Small-world properties were only observed for the French pig trade network, but all networks were more clustered than 100 simulated random networks of the same size (Table 5).

### Trade Communities

Trade networks were divided into 174, 842, 3,070, and 4,362 communities in Bulgaria, France, Italy, and Spain, respectively. The communities were more isolated, i.e., had fewer pig batches moved to or from premises of other communities, in Bulgaria than in the other countries (*w*_c_/*w*_ic_ = 107, 5.7, 6.8, and 6.9 in Bulgaria, France, Italy, and Spain, respectively). Fourteen, 15, 15, and 9 large communities were identified according to the distribution of community sizes in Bulgaria, France, Italy, and Spain, respectively. They included 37.7% (Bulgaria), 51.6% (France), 15.6% (Italy), and 13.7% (Spain) of active premises.

Based on the distribution of the production types and housing system (Figures S5 and S6 in Supplementary Material), three types of production systems could be defined: (i) *type 1 – intensive*: more than 50% of premises were commercial pig farms and <10% raised pigs outdoor; (ii) *type 2 – commercial outdoor*: more than 50% of premises were commercial pig farms and more than 10% raised pigs outdoor; and (iii) *type 3 – small-scale*: more than 50% of premises were small-scale pig farms, raising pigs indoor or outdoor. Only two of the largest communities were of *intensive* type in Bulgaria, the other being of *small-scale* type. In France and Spain, most of the largest communities were *intensive*, except five communities that were of *commercial outdoor* type. They were located in south-western, center, and eastern regions of France and in Extremadura and south of Castille y Leon in Spain. In Italy, only three of the largest communities were of *intensive* type and were located in Lombardia and Piemonte. The others were of *small-scale* type and were located in center and southern regions of Italy.

**Figure 3:**
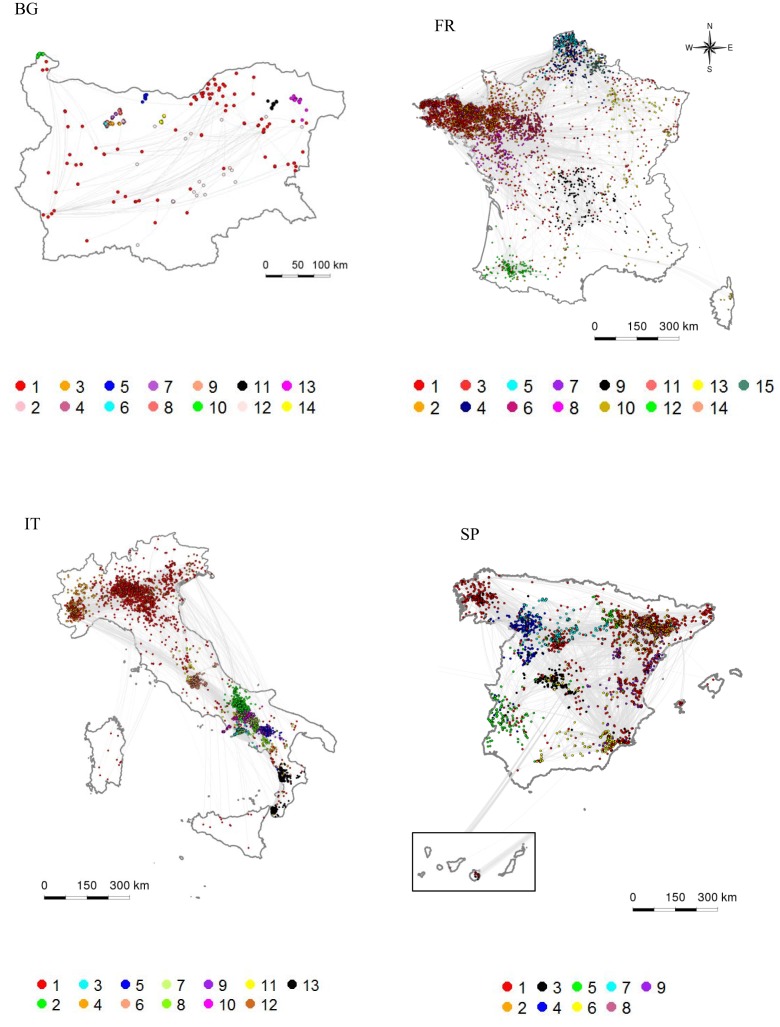
**Spatial and structural characterization of the largest trade communities**. The coloring indicates the community membership, the communities being numbered from the largest to the smallest. (BG: Bulgaria, FR: France, IT: Italy, SP: Spain).

All communities formed spatial clusters, which tended to cover quite large areas and to overlap when the production system was *intensive*, but were highly spatially clustered when it was *small-scale* (Figure 3; Figures S5–S7 in Supplementary Material). All communities were scale-free with average power law scaling parameters comprised between 2.1 and 7.5. All communities of *intensive* type that included trade operators exhibited small-world properties (Table 6; Figures S5–S8 in Supplementary Material). The other communities with small-world properties were two communities of SP that included trade operators in Italy (Communities ID 6 and 10). Communities of *small-scale* type exhibited a star-topology type, reflected by a null clustering coefficient and an average path length of 1 (Table 6). These communities usually consisted of a commercial farm that sent pigs to SP (Figure S9 in Supplementary Material).

**Table 6:** **Statistical properties of selected pig trade communities with different pig production systems from four European countries in 2011**.

Com ID	Region (NUTS 2 level)	No of nodes	% SP	No of TR	% outdoor	CC	AvPL	γ_o_	γ_i_
**Bulgaria**									
1	Severen tsentralen	102	47.1	0	NA	0.094	1.24	2.13	3.54
7	Severozapaden	27	88.9	0	NA	0.000	1.00	2.61	1.88
12	Stara Zagora	24	20.8	0	NA	0.150	1.54	2.26	1.87
**France**									
1	Brittany	2,298	0.1	5	2.5	0.063	2.04	4.51	2.67
4	Nord	310	0.0	5	2.3	0.337	2.15	2.81	3.31
12	Aquitaine/Midi-Pyrénées	153	0.0	0	15.7	0.190	1.97	4.46	2.38
**Italy**									
1	Lombardia	2,025	20.9	6	2.2	0.173	3.99	2.10	4.01
5	Basilicata	439	92.1	0	NA	0.000	1.00	5.80	1.00
13	Calabria	329	79.3	2	11.6	0.000	1.00	5.29	1.00
**Spain**									
1	Galicia/Aragón	1,487	1.2	7	1.2	0.083	2.83	4.74	3.22
4	Castilla y León	257	4.3	2	12.8	0.016	1.10	4.08	2.43
5	Extremadura	248	0.0	0	28.2	0.096	1.36	2.61	3.44

*“Com” represents the largest communities, SP are small producers, TR are trade operators, CC is the clustering coefficient, AvPL is the average path length, and γ_o_ and γ_i_ are the power-law scaling parameters for out- and in-degree distributions, respectively. NA, not applicable*.

## Discussion

This study provides a better understanding of the pig trade structure and characteristics in the EU under diverse production systems, including intensive, commercial outdoor, and small-scale. We also provide valuable proxies for pig movement patterns at country and community levels that can be used to better parameterize more realistic epidemic models under diverse epidemiological scenarios. Results also improve our understanding of trade drivers by highlighting similarities and differences in the functional and spatial organization of pig trade between countries and between production systems.

One of the challenges of this study was to identify and describe European pig production systems, which may have different trading patterns and thus different behaviors regarding infectious diseases but can coexist within a country. The use of the Walktrap community detection algorithm appeared to be a powerful tool as it was able to identify trade communities that match known production systems and areas. For example, intensive production systems in north-west of France, north-east of Spain, and north of Italy were clearly identified. Similarly, the commercial outdoor production systems in Extremadura – Iberian pigs – or in south-west of France, and the small-scale pig production systems in center and southern Italy were also identified. Considering that the movement of animals is the main source of disease introduction/spread into new areas, the use of these methods may help to more cost-effectively trace the sources of infection in case of an epidemic and define zones or compartments that prevent the spread of infectious diseases while maximizing business continuity.

In counterpart, as Walktrap algorithm was used only on live animal movements, small-scale production systems that have or report few exchanges of pigs, such as the Corsican, Sardinian, or the East Balkan pigs, will not emerge. Moreover, in these small-scale production systems, the role of contaminated fomites in the spread of infectious diseases may be more important than the movement of live animals compared to intensive farms, given the absence or low biosecurity levels. Therefore, other methods, such as farmer interviews, should be used to complement the information regarding trade patterns and to identify and describe other high-risk contacts associated with fomites (e.g., vehicles, people, hunting practices, etc.) (46, 47).

In addition, reader should notice that Walktrap algorithm treats directed networks as undirected. Unfortunately, the few algorithms that implicitly consider directionality of the movements (e.g., InfoMap) are computationally intensive and usually do not work for large networks. For example, we were able to use InfoMap for Bulgaria, but the algorithm was not working for France, Italy, or Spain. Nevertheless, for Bulgaria, we had a good agreement using both methods: the number and characteristics of the communities were similar when comparing InfoMap and Walktrap algorithm (i.e., Walktrap: 168 communities, 4 largest 107, 102, 54, and 41 nodes; Infomap: 160 communities, 4 largest: 172, 126, 54, and 54 nodes). Therefore, we assumed that Walktrap algorithm was performing well and was a good choice in this case to describe the modularity of our directed networks.

Report of movements is quite similar and mandatory in the four countries included in the study since at least 2009, and the official veterinarians were quite confident of the reporting compliance for the year 2011, except for some specific areas of the Islands of France and Italy (i.e., Corsica and Sardinia Island). It is important to note that although authorities are usually not very open to share animal movement records with this level of detail (i.e., at farm level and without some temporal or spatial aggregation by month/year or county/region) due to confidentiality issues, there is an extraordinary value of accessing and analyzing this information to unravel the complexity of the trade network structure and characteristics and better inform policies. In fact, thanks to the high quality of movement data and the availability of full datasets from four European countries during the same period, several network measures and proxies of pig trade patterns could be computed and compared in detail. Results highlighted that some proxies can be used whatever the systems considered, whereas other are specific to a country or even a production system (Tables 1–5). Indeed, the scale-free topology was observed for every trade network, whatever the country or the production system considered, as previously reported in countries with a predominantly intensive pig production system (41, 48–51). This means that most premises have few connections while few premises have many connections (38). These premises were mostly trade operators and multipliers but other production types, such as farrow-to-finish or finishers, could also have a lot of connections (Figure S4 in Supplementary Material). They may play an important role in the spread of infectious diseases and could be targeted to more efficiently detect and control them (52). A closer look at the degree distributions also revealed that trade operators behaved differently according to the production system, acting as collectors in industrial systems, and as dispatchers in countries with a lot of SP. Trade operators may thus play different roles as “super-receivers” or “super-spreaders” in disease epidemics and may be good candidates to target risk-based surveillance or control strategies, respectively. Future studies aiming to evaluate weather or not the preferential attachment observed in those scale-free networks can be explained differently in each country will be valuable.

Small-world properties, which had been previously reported for pig trade networks (41, 48, 53), were not observed in this study when considering the whole countries, except for France. They were however observed when considering trade communities, particularly when these communities contained trade operators. Trade operators were present in all communities with indoor commercial producers but were rarely observed in communities mostly comprised of small-scale or outdoor producers. Infectious diseases will thus spread differently by trade movements according to the production system. They will spread quicker, more remotely, and extensively in intensive production systems and slower, more locally, in extensive or small-scale production systems (37, 54). These results suggest that to simulate realistic networks based on network topology (18, 19), modelers should consider that pig trade networks have both scale-free and small-world properties in intensive production systems, but only scale-free properties in outdoor or small-scale production systems. Further analyses using data from other countries could be useful to confirm these results.

Shipment distances were similar between countries, with most of movements occurring within 100 km as previously described in other European countries (26, 49, 55). As expected, the greatest shipment distances where observed in countries with the longest territories (Spain and Italy) and might have been even greater if movements from/to foreign countries had been included in the analysis. However, the distances appeared to be linked with the type productions systems, whatever the country considered (Table 2; Figure S2 in Supplementary Material). The contrast was particularly marked in Bulgaria and Italy, with short shipment distances for communities of small-scale producers and long distances for communities with commercial farms (Figure S7 in Supplementary Material). These short shipment distances might be due to the fact that small-scale producers are usually located in remote areas, such as the less developed areas or mountains, with limited access to expressways or trains. Thus, they tend to trade with neighbors, which are mostly also small-scale producers. They might also be less likely to form connection with geographically and network distant premises, which could explain why the small-world properties were not observed in small-scale production systems. The impact of premises location and transport facilities on shipment distances and network topology could be further investigated to more accurately model pig movements.

For all countries, shipment rates were much lower than those described in recent studies from Canada, even when considering only commercial farms (41, 53). This might be due not only to the higher specialization and inherent more integrated, multi-site, structure of commercial premises in North America (i.e., particularly, US and Canada) but also to differences in data sources and data representativeness and quality as, for example, Dorjee et al. (41) obtained data only from one major pig company and Thakur et al. (53) from volunteer farmers. Shipment rates were particularly low in Bulgaria, illustrating the lower degree of specialization for Bulgarian pig farms, and thus a less need to exchange pigs between premises. This certainly have important implications in terms of disease prevention and control and should be considered when defining zones or compartments to mitigate disease spread while allowing business continuity. Shipment sizes were also not homogeneous between countries. Pig batches sent to finishers were the largest, as previously observed (41, 49); however, those sent to finishing farms in Spain were larger than those sent to finishing farms in France or Italy, likely due to the differences in farm sizes. As expected, pig batches sent from or to SP were of small size, whatever the country considered.

Mixing patterns by premise types are also useful to more realistically simulate pig trade networks (11, 14, 15). Commercial pig production is usually considered to have a pyramidal organization with multipliers sending reproductive pigs to farrowing or farrow-to-finish farms and these ones sending piglets to finishers [e.g., Ref. (26, 49, 50)]. Mixing patterns measured in this study do partially reflect this organization but also highlight some unexpected trade patterns. Indeed, results reveal the major role played by trade operators in France, which tended to proportionally receive most of shipments no matter the type of farm sending pigs, whereas in Spain or Italy, multipliers also played a central role in the pig trade organization. They also highlighted that SP were not isolated, and not only receive but also sent pigs to commercial producers. These mixing patterns are thus important to consider for surveillance or control strategies or when modeling disease spread. Thus, even if shipments rates and shipment distances seem to be linked with the type of production of the premises sending and receiving pigs, mixing patterns could depend on economic or organization rules that are country-dependant. Modelers using models based on statistics on shipments rates, shipment distances, and mixing patterns between production types (14–17) should thus consider this information.

Other methods, such as exponential random graph models (ERGMs), have been recently used to better capture the complex topology and mixing patterns of pig trade networks (56). Our study can be used to select ERGM parameters. For example, the existence of long distance shipments suggest that geographical information should be used in ERGMs to adequately capture the spatial patterns of pig trade at country level.

Community detection methods have been suggested as a useful tool to identify compartments or zones that could be used in the design of diseases surveillance and control programs to preserve business continuity and minimize trade disruption (24, 52). Results of this study suggest that, in general, for disease prevention and control, the most cost-effective strategy in intensive production systems would be the compartmentalization, due to the extensive areas covered, whereas for small-scale production systems, such as in southern Italy or in north-western Bulgaria, zoning would be more effective. The specific topology of pig trade in such areas could also be used to implement risk-based interventions for disease prevention or better control in case of an epidemic. Indeed, only few premises create a bridge between communities (e.g., in Bulgaria, there are Type B farms linking Backyard with Type A farms or Type A farms linking Type B with Industrial farms), and these premises could be targeted to implement control and surveillance measures (28, 29).

Results presented in this study have been obtained considering complete pig trade networks of four different EU countries. The aim of this study was to better understand the complex pig network organization, topology, and structure of the most representative pig production systems present in the EU, including small-scale and outdoor. However, we used data only from 1 year. We do agree that seasonality and reproducibility of results over different years is the key to be able evaluate the validity of our results and its usefulness to inform disease spread models and risk-based interventions (57). Those aspects might be particularly sensible in small-scale production systems where production is known to be seasonal (46). For that reason, we did check the reproducibility between years with some additional information we had available for France, Italy, and Bulgaria (for Spain, unfortunately we did not have multiple years of data available), and we found that results were similar among years for the following parameters: shipment sizes, distances, contact matrix per type of premises, and network topology (data not shown). The largest communities selected in this paper were also stable over the years, covering globally the same geographical area in the different years (although we did not check the percentage of premises belonging to each community for each different year). We also observed that industrial premises did not show a strong seasonality, whereas in small-scale pig production systems (e.g., some parts of Italy and Bulgaria), pig movements had strong seasonal variations clustered in specific time periods but with seasonal patterns repeated yearly (e.g., before Easter and Christmas). Therefore, we believe that even with information from 1 year, results are valuable to inform disease spread models and risk-based interventions. Moreover, most of disease spread models usually inform their parameter values using year-level data (maybe because the use of different parameter values for each month or each season usually increases tremendously the complexity of the model and it can be overwhelming for sensitivity analysis). Nevertheless, other studies should be conducted to address more in detail the seasonality and temporal patterns and characteristics of the pig movement network of different production systems. Particularly, we recommend to explore whether or not the frequency of shipments, the geographical dispersion of the communities and the premises that create bridge between communities are concordant for different years.

## Conclusion

A better understanding of pig trade network patterns, topology, drivers, and characteristics under diverse production systems is the key to more cost-effectively prevent and control endemic and exotic infectious diseases. In this study, we have characterized and compared, for the first time, the pig trade networks in four representative EU countries (Spain, France, Italy, and Bulgaria), which have most of the different productions systems existing in the EU: commercial vs. small-scale and outdoor vs. indoor. Methods and results can be directly used to inform risk-based strategies to better prevent and control future incursions of diseases, such as ASF, CSF, or FMD, or to more realistically parameterize simulation models for those and other diseases affecting swine populations.

## Author Contributions

AR, BM-L, and VG designed the study and developed the R codes. AR and BM-L gathered, cleaned, and verified the data. TA, FF, AW-S, and JS-V contributed to the interpretation and critical discussion of the nature, characteristics, and structure of the data for the different study regions. AR carried out the analyses and wrote the draft of the manuscript. All authors participated in the interpretation and discussion of the results, read, edit, and approved the final manuscript.

## Conflict of Interest Statement

The authors declare that the research was conducted in the absence of any commercial or financial relationships that could be construed as a potential conflict of interest.
