# Supplementary material for: Spatial and Functional Organization of Pig Trade in Different European Production Systems: Implications for Disease Prevention and Control
Source: Front Vet Sci. 2016 Feb 4;3:4. doi: 10.3389/fvets.2016.00004 (PMC4740367; doi:10.3389/fvets.2016.00004)

Figure S 1. Distribution of yearly rates of ingoing and outgoing pig shipments per premise type in four European countries using violin plots (i.e., a combination of boxplots and kernel density plots showing the distribution of the variable). Y-axis is in log-scale. Only active premises were considered.

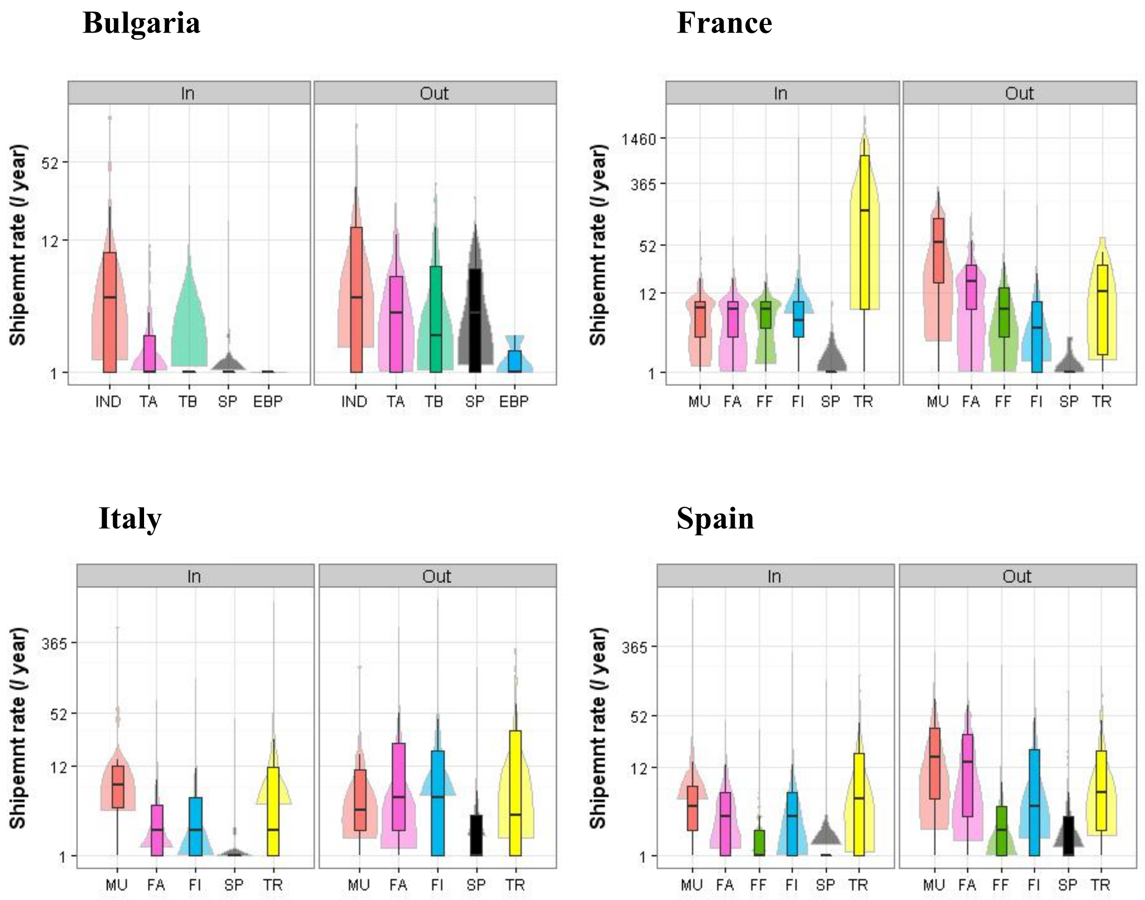

Supplement: Supplementary file 1 [file Image_1.PDF]
