# Supplementary material for: Spatial and Functional Organization of Pig Trade in Different European Production Systems: Implications for Disease Prevention and Control
Source: Front Vet Sci. 2016 Feb 4;3:4. doi: 10.3389/fvets.2016.00004 (PMC4740367; doi:10.3389/fvets.2016.00004)

Figure S 3. Distribution of the shipment size per premise type pairs in several European countries in 2011 using violin plots (i.e., a combination of boxplots and kernel density plots showing the distribution of the variable; Premise type: IND = industrial farm; TA = type A farm; TB = type B farm; SP = small producer; EBP = East Balkan Pig farm; MU = multiplier; FA = farrowing farm; FF = farrow-to-finish farm; FI = finishing farm; TR = trade operator).

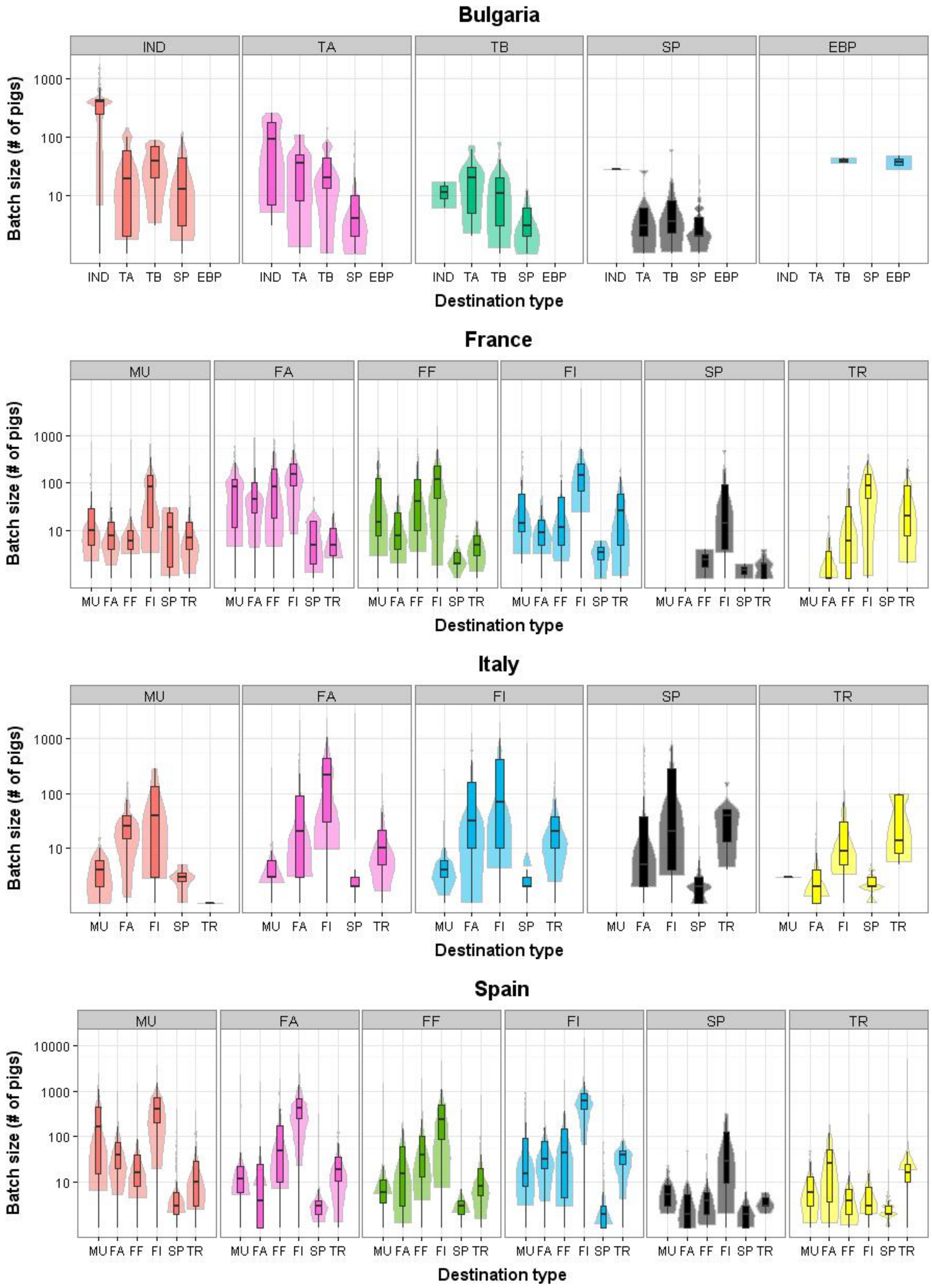

Supplement: Supplementary file 3 [file Image_3.PDF]
