# Supplementary material for: Spatial and Functional Organization of Pig Trade in Different European Production Systems: Implications for Disease Prevention and Control
Source: Front Vet Sci. 2016 Feb 4;3:4. doi: 10.3389/fvets.2016.00004 (PMC4740367; doi:10.3389/fvets.2016.00004)

Figure S 7. Distances between pairs of pig premises belonging to the largest trade communities in Bulgaria, France, Italy and Spain in 2011 (the colors correspond to those used in figure 3 of the manuscript)

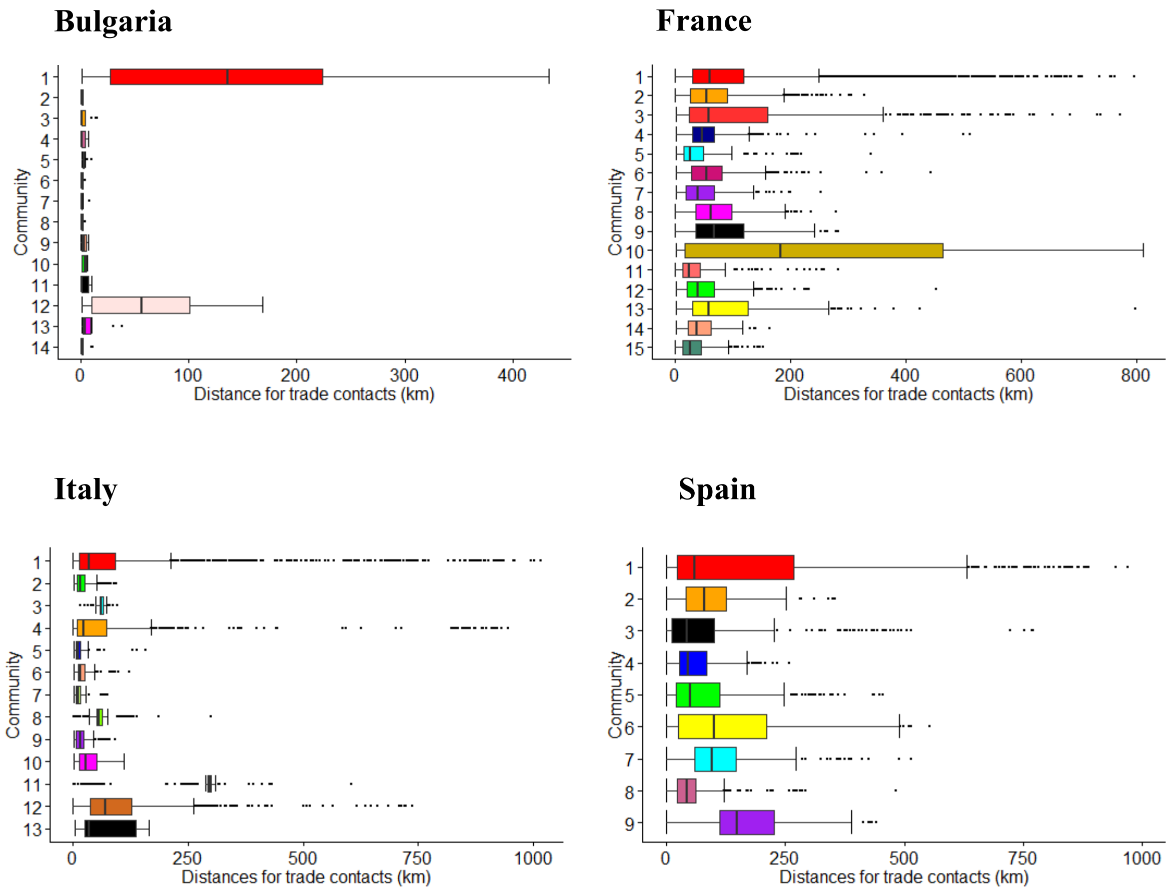

Supplement: Supplementary file 7 [file Image_7.PDF]
